# 2395: Self-assembling cartilage from equine mesenchymal stem cells: A comparison of bone marrow and cord blood-derived MSCs

**DOI:** 10.1017/cts.2017.46

**Published:** 2018-05-10

**Authors:** Jamie L. White, Jerry C. Hu, Dori L. Borjesson, Kyriacos A. Athanasiou

## Abstract

OBJECTIVES/SPECIFIC AIMS: Joint injury is a common cause of premature retirement for many equine athletes. Implantation of engineered cartilage offers the potential to increase the success rate of surgical intervention and hasten recovery times. Mesenchymal stem cells (MSCs) offer a particularly attractive cell source for cartilage engineering. Although bone marrow-derived MSCs (BM-MSCs) have been most extensively characterized for musculoskeletal tissue engineering, studies suggest cord blood MSCs (CB-MSCs) may elicit a more robust chondrogenic phenotype. The objective of this study was to determine superior equine MSC source for cartilage engineering via a self-assembling process (SAP). METHODS/STUDY POPULATION: MSCs derived from bone marrow or cord blood were stimulated to undergo chondrogenesis via 3D culture and then used to generate cartilage via SAP. The resulting neocartilage produced from either BM-MSCs or CB-MSCs was compared by measuring biochemical, mechanical, and histological properties. RESULTS/ANTICIPATED RESULTS: We found that while BM-MSCs possessed higher tensile properties and collagen content, CB-MSCs had superior compressive properties and GAG content. Moreover, CB-MSCs had lower alkaline phosphatase activity and higher collagen type II, suggesting a more hyaline cartilage-like phenotype. DISCUSSION/SIGNIFICANCE OF IMPACT: In conclusion, while both BM-MSCs and CB-MSCs were able to form neocartilage, CB-MSCs resulted in tissue more closely resembling native equine articular cartilage, and is therefore the superior MSC source for purposes of cartilage self-assembly.

